# Enhancing Students’ Self-Efficacy in Creativity and Learning Performance in the Context of English Learning: The Use of Self-Assessment Mind Maps

**DOI:** 10.3389/fpsyg.2022.871781

**Published:** 2022-05-11

**Authors:** Zi Yan, John Chi-Kin Lee, Sammy King Fai Hui, Hongling Lao

**Affiliations:** Department of Curriculum Instruction, The Education University of Hong Kong, Hong Kong, Hong Kong SAR, China

**Keywords:** creativity, self-efficacy in creativity, English language learning, self-assessment, mind mapping, experimental design

## Abstract

Creativity is an important 21st Century skill that enhances students’ ability to see new opportunities, confront new challenges, and adapt flexibly to the changing study, work and life situations. To nurture students with strong self-efficacy in creative thinking is as important as the contexts and strategies involved in its application. But how to develop sustainable interventions (without generating excessive workload for teachers) to promote students’ self-efficacy in creativity is a long-lasting challenge. This study presents a simple and relatively cost-effective instructional intervention, i.e., self-assessment mind maps, and examines its effect on students’ self-efficacy in creativity, self-efficacy in learning English, and academic performance in English language tests. A pretest-posttest non-equivalent design was adopted for the experimental and control groups of students in a Hong Kong primary school in 2021/22 Spring semester. The results show that students from the experimental group significantly outperformed those from the control group on self-efficacy in creativity after the intervention. However, the intervention did not improve students’ self-efficacy and test performance in English learning. The findings demonstrate the potential of self-assessment mind maps as an effective and sustainable instruction intervention to promote students’ higher-order abilities. This study sheds light on designing sustainable instructional strategies for empowerment in creativity.

## Introduction

Mind mapping is a tool to scaffold visual thinking for and as learning (Buzan and Buzan, 2002, 2006). As mind mapping enables students to freely express ideas and connect those ideas in a non-linear manner, it stimulates divergent thinking (Leeds et al., 2019) and, therefore, has advantages in developing higher-order abilities, such as creativity. Due to its flexibility and simplicity, mind mapping has been widely used in various contexts. Although past studies have revealed a generally positive impact of mind mapping on academic and affective outcomes, the effect varied across contexts (Liu et al., 2014). Thus, further empirical research is needed in this direction to identify effective interventions.

Considering that mind mapping could be cognitively demanding for some students, appropriate scaffolding is necessary. Moreover, reviewing students’ mind maps and providing feedback could be time-consuming for teachers, making it less likely to be sustainable. Thus, it is desirable to design easy-for-use (for students) and cost-effective (for teachers) mind mapping strategies.

This study aims to examine the effectiveness of a simple intervention, i.e., the self-assessment mind map that combines the ideas of self-assessment and mind mapping, on primary school students’ self-efficacy in creativity, self-efficacy in learning English, and academic performance in English language tests in a naturalistic learning environment. The findings can inform the design of sustainable mind mapping interventions to promote students’ higher-order abilities.

### What Is Mind Mapping

A picture is worth a thousand words. While verbal language is ubiquitous in routine teaching and learning, visualization captures less attention regardless of its potential to enhance higher-order abilities and offer an alternative pathway for learning. Mind mapping is such a tool to scaffold visual thinking for and as learning (Buzan and Buzan, 2002, 2006). Different from the strict rules in the language (e.g., grammar), mind maps offer more freedom and flexibility in information processing. Starting with a theme, relevant elements are freely represented in words, symbols, or pictures, reducing linguistic barriers to the thinking process, especially for younger children. The relation among elements is represented with lines, resulting in a network of information on this specific topic. The creation of a mind map is often an impromptu and non-linear process, with a high sense of autonomy and personalization. Due to its flexibility and simplicity, mind mapping has been widely used in various contexts. For example, enterprises can use it for knowledge management, especially in digital form (Lin and Faste, 2011). Professional workers can use it for brainstorming, communication, and planning, especially for people with impaired vision (Schnelle-Walka et al., 2014). Students can use it for note-taking, brainstorming in writing and group projects, as well as reflection (Vijayakumar, 2011; Tee et al., 2014).

From a constructivist perspective, the process of creating elements and making connections requires a deep and active way of information processing (Dhindsa et al., 2011), promoting personal engagement and enjoyment. On the other hand, because of its minimum restriction during creation, mind mapping stimulates divergent thinking instead of convergent thinking (Leeds et al., 2019). Ideas are freely expressed and connected in a non-linear manner without the necessity of following the strict rules as those enforced in writing. It is advantageous in incubating creativity. Furthermore, according to the multiple intelligence theory (Gardner, 1993, 2011), people are born with different talents and tendencies. Some excel in manipulating verbal tools, whereas some are good at thinking with visual elements. Mind mapping offers an alternative way of thinking and understanding in formal education, which could benefit those with high visual-spatial intelligence but low linguistic intelligence.

### The Impact of Mind Mapping

Due to the features elaborated earlier, mind mapping has attracted increasing research interest and past studies have documented the positive impact of mind mapping in enhancing a wide range of desirable learning outcomes, such as higher-order abilities (e.g., creativity), affective outcomes (e.g., self-efficacy in learning), and academic performance. Liu et al.’s (2014) meta-analysis of the effect of mind mapping included 52 studies. They found that, in general, the use of mind maps lead to positive changes in academic and affective outcomes. However, the effect varied across studies and some studies reported nil or even negative impact.

Mind mapping is an effective strategy to develop higher-order abilities, such as creativity. In their review, Wang et al. (2010) concluded that the strategy of mind mapping is helpful for creativity and problem-solving because drawing with words and non-word symbols can trigger students’ learning motivation and evoke their abstract thinking. There is empirical evidence in the literature. For example, Hwang et al. (2012) conducted an experimental study investigating the effect of mind mapping in an undergraduate business planning course. There were two experimental groups: one with a mobile-based mind map application, the other with a computer-based mind map application. The control group adopted the conventional instruction. The results showed that students in both experiment groups (mobile-based and computer-based mind maps) are significantly superior to those from the control group in terms of creative thinking ability. They argued that the graphic representations and drawing in mind maps facilitate students’ engagement in divergent thinking which, in turn, promote creativity. In another experimental study with university students, Malycha and Maier (2017) found that the use of the mind mapping technique significantly enhanced students’ performance on each of the three creativity dimensions (i.e., fluency, flexibility, and originality).

The pedagogical impact of mind mapping on creative thinking has also been studied in the context of learning English, but the results are mixed. For example, Buran and Filyukov (2015) found that mind mapping was helpful for students to brainstorm creative ideas, learn new vocabulary, enhance reading skills, and organize presentations. Another study by Yunus and Chien (2016) revealed that the use of mind maps facilitated students to gain a deeper understanding of the writing topics, plan the writing, and enhance creativity in writing. Fu et al. (2019) designed a mind mapping-based contextual gaming approach and revealed that the new approach enhanced students’ writing performance and ability to generate diverse ideas. In contrast, Ningrum et al. (2016) reported that mind maps did not significantly impact EFL students’ idea development in argumentative writing. Nevertheless, most students had positive perceptions of mind maps and were willing to engage in mind-mapping-based activities (Yunus and Chien, 2016; Fu et al., 2019).

The conception of creativity encompasses a wide range of creative people, processes, and products (Beghetto and Kaufman, 2007). In this study, we assessed how the construct of “creative self-efficacy” varies to capture the magnitude of creativity during the learning process. Rooted in the work of Bandura (1986, 1993), creative self-efficacy, a special form of self-efficacy, is a person’s self-belief and judgment of one’s own ability to be engaged in creative activities (e.g., Tierney and Farmer, 2002; Tang et al., 2017). Self-efficacy contributes to human cognitive, motivational and affective development and functioning (Bandura, 1997). Thus, a strong sense of self-efficacy is a necessary condition for human creative productivity. Creative self-efficacy has a medium-size correlation (*r* = 0.39) with different creativity measurements, as shown in a meta-analysis synthesizing 41 papers (Haase et al., 2018). Many studies in the Asian context focused on teachers’ creativity beliefs (e.g., Huang and Lee, 2015; Huang et al., 2019a,b), but the understanding of students’ creative self-efficacy, particularly its relationship with mind mapping strategies, is limited. This study aimed to address this gap. In this study, creative self-efficacy is expressed as self-efficacy in creativity to stay consistent with the other variable (i.e., self-efficacy in learning English).

The use of mind maps can improve knowledge retention and clarify concepts that lead to an in-depth understanding of learning contents and deeper learning among students (Bressington et al., 2018). Thus, the repeated engagement in mind maps can offer students a sense of encouragement and success, leading to enhanced students’ self-efficacy (Nesbit and Adesope, 2006). Past studies have also investigated the impact of mind mapping on students’ self-efficacy in learning, but the findings are mixed. Some studies found that using mind maps in teaching could increase students’ self-efficacy in learning (e.g., Chularut and DeBacker, 2004; Zheng et al., 2020), while the others reported non-significant results. For instance, Bressington et al. (2018) found in their experimental study that students from the experimental group (using mind maps) showed lower self-efficacy than those from the control group (conventional teaching approach), although the difference was not statistically significant.

In addition to self-efficacy, mind mapping has been approved to be a useful learning and teaching tool to enhance students’ academic performance in various subject areas, such as Programming (e.g., Gul et al., 2017), Economics (e.g., Madu and Metu, 2012), and learning English (e.g., Wang, 2019; Hazaymeh and Alomery, 2022). Hazaymeh and Alomery (2022) found that mind mapping, using the web-based mind mapping software “MindMeister,” helped improve English language learners’ reading ability in an online learning environment. In learning English grammar, mind mapping helps students organize the knowledge points in a systematic and visual fashion that is useful to deepen their comprehension of the knowledge points and the connection between them (Wang, 2019). Such a positive impact of mind mapping is also applied to young EFL learners. Lan et al. (2015) reported that mind mapping significantly improved fifth graders’ grammar knowledge compared to those who did not use this strategy. Merchie and van Keer (2016) investigated the effect of two instructional mind mapping strategies (researcher-provided or student-generated mind maps) on fifth and sixth graders’ graphical summarization skills. They found that students from the experimental group outperformed those from the control group on most, but not all, aspects of graphic summarization skills. Furthermore, student-generated mind maps showed a stronger impact than researcher-provided mind maps. However, some studies reported unfavorable results. Ritchie et al. (2013) conducted two experiments to test the effect of mind mapping on primary school students’ learning. Although they found a significantly positive impact of mind mapping on the retrieval practice in Experiment 1, they did not find any significant main or interaction effects in Experiment 2, which had a larger sample.

### Combining Mind Mapping and Self-Assessment

The design and implementation of mind maps are associated with some challenges. In many cases, it is time-consuming for students to produce mind maps and for teachers to review mind maps. Furthermore, as mind mapping requires students to recall, organize, and visualize their cognitive structures, some students may find it cognitively demanding and need additional scaffolding (Stokhof et al., 2020). There are suggestions to provide students with pre-set templates (Prabha and Aziz, 2020) or worked-example mind maps (Merchie and van Keer, 2016) to facilitate the generation of mind maps and maximize its impact on student learning.

As producing mind maps relies on students’ reflection on the learning process they have experienced, scaffolds that facilitate self-reflection would be useful. In this sense, it is promising to combine mind mapping and self-assessment in instructional design. Self-assessment refers to “a process during which students collect information about their own performance, evaluate and reflect on the quality of their learning process and outcomes according to selected criteria to identify their own strengths and weaknesses” (Yan and Brown, 2017, p. 1248). In the self-assessment process, students are encouraged to take responsibility for their learning by acting as not only recipients of assessment but also designers and users of assessment (Wu et al., 2021b). Therefore, self-assessment is a learning process, rather than an assessment method, that provides students with ample learning opportunities (Yan and Carless, 2021).

Past studies have shown that students’ engagement in self-assessment can lead to improved academic performance (Brown and Harris, 2013; Yan et al., 2021). Self-assessment can also increase students’ self-efficacy (Panadero et al., 2017). This was because self-assessment can provide students with a better understanding of the gap between their current and desirable performance levels and adaptive strategies to close the gap. Thus, students are likely to perform better and the successful experience, according to social cognitive theory (Bandura, 1986), increase the perceived capability which, in turn, results in a higher level of self-efficacy. There is also a positive link between self-assessment and creativity. In a recent review, Bolden et al. (2020) concluded that self-assessment or self-reflection could promote both creative products and processes. This was because self-assessment encouraged students to reflect on their learning process and products against assessment criteria. Such reflective thinking could result in adaptive learning strategies or innovative pathways to the learning goals. For example, students who were supported to self-assess or self-reflect demonstrated higher levels of creative perceptions (Eow et al., 2010) and divergent thinking skills (Doron, 2017). Kim et al. (2016) examined the relationship between self-reflection and creativity in the context of English learning. Students used visual thinking to represent their understanding of curriculum content. Students in the experimental group who were supported via tablet technology to reflect on and adjust their visual representations demonstrated significantly higher creativity scores than the control group.

By highlighting students’ active and reflective role in the assessment process, self-assessment enhances students’ agency in learning and avoids the constraints associated with teacher-directed assessment (e.g., big class size and teacher workload) and, therefore, makes self-assessment-based instruction more likely to be sustainable (Yan and Brown, 2021). More importantly, the self-assessment process has been unpacked as concrete and sequential actions (e.g., Yan and Brown, 2017) so that it is possible to scaffold students’ self-assessment in a visual approach, i.e., in the mind map format. Thus, we designed a self-assessment mind map in this study to synergize the impact of mind mapping and self-assessment. On the one hand, the self-assessment framework can provide a generic structure as additional scaffolding for students who were new to mind mapping, making the mind maps concrete enough for easy implementation without burdening teachers. On the other hand, the visual approach of mind mapping makes self-assessment more interesting. Therefore, it could help develop students’ self-assessment ability during the mind mapping process.

### The Current Study

The current study aimed to examine the effectiveness of self-assessment mind maps on students’ self-efficacy in creativity, self-efficacy in learning English, and academic performance in English language tests. We hypothesize that the use of self-assessment mind maps in an English course can enhance students’ self-efficacy in creativity (H1), self-efficacy in learning English (H2), or academic performance in English language tests (H3).

## Materials and Methods

### Participants

A total of 55 students participated the study (male = 32, female = 23, *M*_age_ = 9.26, *SD*_age_ = 0.63, age-range = 8–11 years, grade = primary 4). One class with 24 students was randomly assigned to the experimental group, whereas the other class from the same grade with 31 students was assigned to the control group. The two classes were taught by the same teacher. Four students from the experimental group were excluded from analysis due to the lack of either posttest or post-survey data. As a result, the valid sample size for this study was 51 (experimental = 20, control = 31).

### The Design and Procedure

**FIGURE 1:**
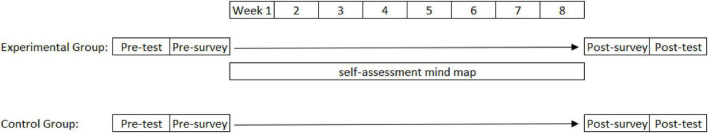
The research procedure.

A quasi-experimental design with intact classes in naturalistic settings was applied. The two classes were randomly assigned one of the two conditions: self-assessment mind map group (the experimental group) or no mind map group (the control group). The research procedure is presented in Figure 1.

Students’ self-efficacy in creativity and self-efficacy in learning were assessed before and after the intervention. The pre-intervention survey was administered 2 days before the intervention, and the post-intervention survey was administered 3 days after the intervention. To minimize the teacher’s and students’ workload, we used students’ performance on the school exams as the indicator of their academic performance. The exam used as the pretest was administered 2 weeks before the intervention, and the exam as the posttest was administered 1 week after the intervention. All participants received the same instruction from the same teacher during the 8-week intervention. Students in the experimental group were asked to complete the self-assessment mind map twice a week. In total, students completed 16 mind maps during the intervention. In contrast, students in the control group were not required to complete the mind map. To minimize the teacher’s workload, the teacher was not asked to review nor provide feedback on students’ mind maps. To reduce the possible noise to the experimental results, such as *Rosenthal effect* (Rosenthal and Jacobson, 1992), the research team told the teacher that this study is exploratory and the impact of the intervention is unknown.

This study was approved by the ethical review committee of the author’s affiliated university. All participants, including the teacher, students and their parents/guardians, signed written consent forms.

### Measures

#### English Language Tests

For the sake of reducing workload for both the teacher and students, we used students’ scores on the school exams as the indicator of their academic performance instead of using specifically designed tests.

#### Self-Efficacy in Creativity

Creative self-efficacy was measured by the scale *creative self-efficacy in English*, adapted from Beghetto et al. (2011). To better detect changes, creativity was set to be subject-specific rather than general. It was measured by five items (Cronbach α = 0.84), with a sample item as “I have a lot of good ideas during English class.”

#### Self-Efficacy in Learning English

Self-efficacy was measured by the *self-efficacy subscale* from the Motivated Strategies for Learning Questionnaire (MSLQ; Pintrich et al., 1991), with seven items (Cronbach α = 0.86). A sample item was “I am certain that I can understand the ideas taught in class.” The items were designed in general, but the survey instruction was domain-specific. Participants were instructed to respond based on their learning experience in the subject of English. The questionnaire is attached in Supplementary Appendix A (English version) and Supplementary Appendix B (Chinese version).

### The Self-Assessment Mind Map

A semi-structural mind map template was designed to maximize its learning benefits while minimizing teacher workload. The template integrated the self-assessment process model (Yan and Brown, 2017) as its generic structure across lessons, while maintaining its flexible and autonomous feature in content and format. Yan and Brown (2017) specified the self-assessment process into concrete and sequential actions. When engaging in self-assessment, students first determine a performance standard against which they will evaluate their learning process and outcomes in a lesson (i.e., *determining criteria*). They then reflect upon their learning and identify their strengths and weaknesses (i.e., *self-reflection*). Students may also take the initiative to seek additional resources or feedback for their learning (i.e., *feedback-seeking*) when their perceived learning resources are insufficient.

Corresponding to the three self-assessment steps, guiding questions were provided in the mind map template to facilitate students’ reflection. One prompt (i.e., What is the study theme today?) was used to anchor students’ self-assessment. It was not an assessment *criterion*, but it specified the scope for students’ self-assessment. Two prompts (What have you learnt today? What confuses you?) were used to guide students to have more focused *self-reflection*. One prompt (i.e., Who/What can help you learn?) acted as a stimulus for students’ *feedback-seeking* behavior. In addition to the guiding questions, an exemplar mind map with “animal” as the theme was provided to make students better understand what they were expected to do in the mind map. Two versions of mind map templates with different colors and progressive complexity were used to make them more attractive to students. The first version has four prompts, and the second version added one additional *self-reflection* prompt (i.e., What tips can help you learn?) to stimulate reflection on their learning strategy. The first version was used for the first 4 weeks and the second version for the last 4 weeks. The mind map templates are attached in Supplementary Appendix C.

Apart from the basic structure, its content and format remain open to students, encouraging divergent thinking and creativity. Students at any ability level were free to create a mind map in their own way, simple or complex. Moreover, these mind maps were only used for the formative purpose, neither graded nor accounted for the final score. They were treated as alternative learning opportunities for students with adequate autonomy and minimal supervision.

### Data Analysis

Within-group difference across time was examined using the paired *t*-test for the three outcome variables. To investigate between-group difference, three one-way analyses of covariance (ANCOVA) were conducted for the three outcome variables (Kenny, 1975; Gribbons and Herman, 1996), with pretest scores as covariates to account for the group difference before intervention. This was common in quasi-experimental design while the random assignment of participants was not feasible (e.g., Choi et al., 2014; Agboghoroma, 2015; Reeves et al., 2017). SPSS 27.0 was used for all analyses.

## Results

### Scale Quality

We examined the quality of scales used in this study before the primary analyses. Both the *self-efficacy in creativity* and *self-efficacy in learning English* scales had high reliability coefficients (see Table 1). The confirmatory factor analyses showed a good fit for both scales (see Table 1). All items loaded onto their respective latent constructs with factor loadings ranging from 0.404 to 0.865. All loadings were significant at *p* < 0.05. The item correlations and item-level statistics for both scales, which are available in Supplementary Appendix D, also demonstrated the high quality of the scales.

**TABLE 1:** Goodness of fit in confirmatory factor analyses.

Scale	#	α	SRMR	CFI	TLI	RMSEA	χ ^2^	df	*p*
Self-efficacy in creativity	5	0.89	0.050	0.995	0.991	0.047	5.60	5	0.347
Self-efficacy in learning English	7	0.86	0.053	0.983	0.968	0.070	13.86	11	0.241

### Within-Groups Comparisons

**FIGURE 2:**
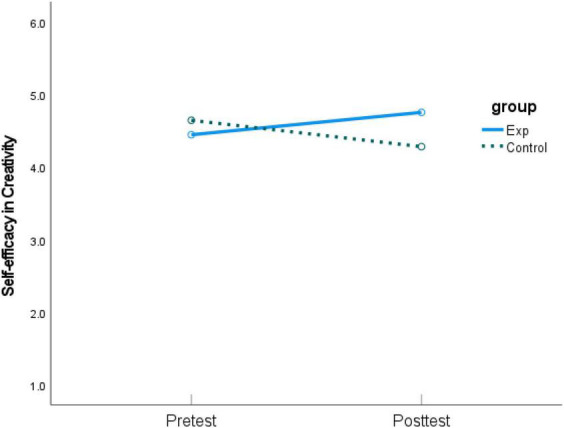
Means of pretest and posttest on *self-efficacy in creativity*.

**FIGURE 3:**
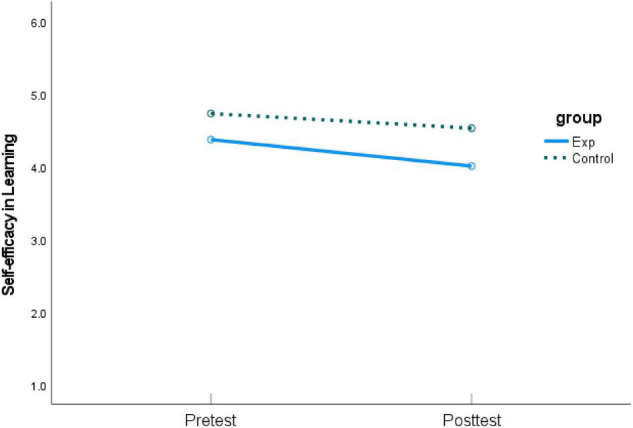
Means of pretest and posttest on *self-efficacy in learning English.*

**FIGURE 4:**
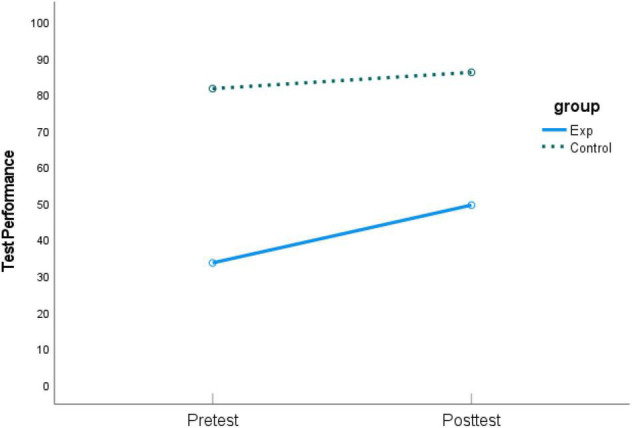
Means of pretest and posttest on *English language test performance.*

As shown in Table 2, students from the experimental group revealed a positive but non-significant change after the intervention for *self-efficacy in creativity*, whereas those from the control group had a significant decrease over time. As for *self-efficacy in learning English*, both groups decreased over time, with the experimental group showing a significant drop. Furthermore, compared with pretest, both groups significantly increased their *English language test performance* at posttest. The experimental group showed a larger gain than the control group, which had a lower pretest mean score. Graphic comparisons between pretest and posttest of the three dependent variables are shown in Figures 2–4.

**TABLE 2:** Pretest-posttest comparisons on learning outcomes.

		Pretest		Posttest		Mean difference			
Measure		*M*	*SD*	*M*	*SD*	*M*	*SD*	*t*	*p*
Self-efficacy in creativity	E	4.47	1.13	4.75	0.98	0.23	1.09	0.93	0.36
	C	4.63	0.93	4.28	1.18	–0.37	0.89	−2.33*	0.03
Self-efficacy in learning English	E	4.37	1.05	4.01	1.04	–0.38	0.76	−2.23*	0.04
	C	4.71	0.68	4.53	0.84	–0.17	0.67	–1.40	0.17
English language test performance	E	33.44	13.95	49.39	14.81	15.94	9.08	7.45**	0.00
	C	81.23	7.62	86.03	6.36	4.50	8.58	2.88*	0.01

E standards for experimental group (n = 20); C stands for control group (n = 31); * is significant at 0.05 level; ** is significant at 0.01 level.

### Between-Groups Comparisons

Between-group differences were examined with one-way ANCOVA on posttest of *self-efficacy in creativity, self-efficacy in learning English*, and *English* language *test performance*, respectively, with pretest scores used as covariates to account for between-group differences before intervention. The assumption of the equality of error variances was checked via Levene’s Test, and the data did not violate this assumption (*p* = 0.61, 0.65, 0.11, respectively). Moreover, the assumption of parallel lines was checked by adding an interaction term between group and pretest score (Keppel, 1991). Results showed that the interaction term was non-significant for both *self-efficacy in creativity* (*F*[47,1] = 2.98, *p* = 0.091) and *self-efficacy in learning English* (*F*[47,1] = 0.07, *p* = 0.791), but significant for *English language test performance* (*F*[49,1] = 8.59, *p* = 0.005). Accordingly, the interaction term was removed for the first two ANCOVA models, but kept and interpreted for the third one.

As shown in Table 3, for *self-efficacy in creativity*, the main effect of group was significant (*F*[1, 48] = 4.30, *p* = 0.04), indicating a significant difference between the posttest scores in the experimental group and the control group after controlling for pretest scores. As shown in Table 4, the parameter for group was 0.54 (*p* = 0.04), indicating the posttest score of *self-efficacy in creativity* for the experimental group was 0.54 higher than that for the control group after controlling for the pretest scores, with a medium effect size ($\eta_{\text{p}}^{2}$ = 0.08) (Olejnik and Algina, 2000), supporting H1. The results in Table 5 showed that the main effect of group was non-significant for *self-efficacy in learning English* (*F*[1,48] = 2.45, *p* = 0.12), indicating the non-significant difference between the posttest scores after controlling for pretest scores. Thus, H2 was not supported. The results in Table 6 showed that the interaction term between group and pretest was significant for *English language test performance* (*F*[49,1] = 8.59, *p* = 0.005), indicating the effect of intervention varies based on different pretest scores. The main effect of group was significant for *English language test performance* (*F*[1,49] = 10.50, *p* = 0.002), indicating a significant intervention impact for students with average pretest scores. As shown in Table 4, the impact is negative (*b* = −47.24 while pretest = 69.89), not supporting H3. However, as shown in Table 2 and Figure 4, both groups improved in the *English language test performance* at posttest, and students in the experimental group exhibited a larger gain in posttest scores than students in the control group. The difference in slope may be due to a ceiling effect for the control group, resulting in a narrower range of improvement. In summary, given the impact of the intervention on *English language test performance* was conditional on pretest scores, this finding remains inconclusive for H3.

**TABLE 3:** One-way ANCOVA on *self-efficacy in creativity* between two groups.

	Sum of Squares	df	Mean Square	*F*	*p*	$\eta_{\text{p}}^{2}$
Intercept	6.07	1	6.07	7.42	0.01*	0.13
Pretest	19.95	1	19.95	24.37	0.00**	0.34
Group	3.52	1	3.52	4.30	0.04*	0.08
Error	39.28	48	0.82			

R Squared = 0.362 (Adjusted R Squared = 0.335); * is significant at 0.05 level; ** is significant at 0.01 level.

**TABLE 4:** Summary of parameters of one-way ANCOVA on three outcome variables.

	Parameter	*B*	Standard error	*t*	*p*	$\eta_{\text{p}}^{2}$
Self-efficacy in creativity	Intercept	1.35	0.61	2.20	0.03*	0.09
	Pretest	0.63	0.13	4.94	0.00**	0.34
	Group	0.54	0.26	2.07	0.04*	0.08
Self-efficacy in learning English	Intercept	1.19	0.55	2.17	0.03*	0.09
	Pretest	0.71	0.11	6.27	0.00**	0.45
	Group	–0.31	0.20	–1.57	0.12	0.05
English language test performance	Intercept	69.89	13.99	5.00	0.00**	0.89
	Pretest	0.20	0.17	1.16	0.25	0.45
	Group	–47.24	14.58	–3.24	0.00**	0.33
	Group*Pretest	0.60	0.21	2.93	0.01*	0.18

* is significant at 0.05 level; ** is significant at 0.01 level.

**TABLE 5:** One-way ANCOVA on *self-efficacy in learning English* between two groups.

	Sum of squares	df	Mean square	*F*	*p*	$\eta_{\text{p}}^{2}$
Intercept	1.75	1	1.75	3.91	0.05	0.08
Pretest	17.61	1	17.61	39.30	0.00*	0.45
Group	1.10	1	1.10	2.45	0.12	0.05
Error	21.50	48	0.45			

R Squared = 0.496 (Adjusted R Squared = 0.476); * is significant at 0.05 level.

**TABLE 6:** One-way ANCOVA on *English language test performance* between two groups.

	Sum of squares	df	Mean square	*F*	*p*	$\eta_{\text{p}}^{2}$
Intercept	2059.34	1	2059.34	40.26	0.00**	0.89
Pretest	1207.42	1	1207.42	23.61	0.00**	0.45
Group	536.91	1	536.91	10.50	0.00**	0.33
Group*Pretest	439.62	1	439.62	8.59	0.01*	0.18
Error	2506.31	49	51.15			

R Squared = 0.888 (Adjusted R Squared = 0.881); ** is significant at 0.01 level.

## Discussion

This study designed a simple, relatively cost-efficient instructional intervention, i.e., the self-assessment mind maps, and examined its effect on students’ self-efficacy in creativity, self-efficacy in learning English, and academic performance in English language tests. The most promising finding is that students from the experimental group had a positive change in self-efficacy in creativity after the intervention, whereas those from the control group experienced a significant decrease over time (see Figure 2). The difference between the experimental and control group was statistical significance after controlling for pretest difference, supporting H1. This finding is consistent with previous studies (e.g., Wang et al., 2010; Hwang et al., 2012; Malycha and Maier, 2017) that reported a positive impact of mind mapping on students’ higher-order abilities, and provides further credits to the mind mapping strategy as we focused on an understudied outcome, i.e., self-efficacy in creativity. Mind mapping as a form of visual technique enhances the processing depth of the subject matter and links diverse aspects to each other in a meaningful and constructive way (Malycha and Maier, 2017). As explained by Liu et al.’s (2014) meta-analysis of 52 studies, referring to cognitive load theory (Miller, 1956; Sweller et al., 1998), mind maps enable students to acquire knowledge easier and clearer, reduce their working load, and allow them to use higher-level schemas in an active and constructive way for the development of intellectual skills. Self-assessment mind-maps, which this study introduced in English language learning, provided personal and contextual sources for students’ creative self-efficacy formulation (Gist and Mitchell, 1992) and deepened students’ beliefs about their ability to engage in creative activities. It is possible that the minimum constraints on the creation process evoke divergent thinking and unleash creativity. In addition, the flexibility and autonomy granted by the creation process, along with the absence of summative consequences in its product, cultivate a sense of control in students, thus improving their self-efficacy. With a stronger sense of self-efficacy in creativity, students are more likely to attribute their success in creative activities to the personal efforts they exerted.

The increase of experimental group students’ self-efficacy in creativity can also be explained by their performances in the mind maps. Firstly, the artistic elements increased in their mind maps. At the beginning of the intervention, all students used words exclusively to express their ideas. However, starting from the 3rd week, students had increasingly mixed text, symbols, and pictures in their mind maps. Past studies showed that the use of artistic elements in mind maps, such as combining text and picture, was related to a higher level of originality and creativity (Mento et al., 1999; Dong et al., 2021). The use of various colors, pictures, or words in mind maps not only facilitate thinking and analyzing, but also enhance originality and creativity among children (Wang et al., 2010; Dong et al., 2021). Secondly, the connections between knowledge points in mind maps were becoming increasingly complex with time. In the beginning, students only wrote words or sentences around the first-level concepts (e.g., what have you learnt today? What confuses you?). When students were more familiar with the mind maps, they tended to demonstrate their understanding of the idea hierarchy. For example, for the learning theme “mini writing,” some students proposed “interview” as the first-level concept under which they further proposed “interviewer/interviewee,” or “questioning/answer,” as the second-level concept. In this sense, mind mapping appears as a better strategy to facilitate a more precise understanding of the idea hierarchy and knowledge network (Leeds et al., 2019). With this strategy, students were more likely to generate more profound and unique responses and develop their critical thinking skills (Long and Carlson, 2011).

However, the impact of mind mapping on students’ self-efficacy in learning English and academic performance in English language tests were not significant. The results showed that students in both groups had improved their English language test performances from pretest to posttest. The magnitude of change of students from the experimental groups was larger than that of students from the control group. The experimental group benefited to a great extent from self-assessment mind maps to reach a marginally satisfactory level of academic performance (*M*_*exp*_ increased from 33.44 to 49.39). However, the between-group difference was not statistically significant, not supporting H2. Students’ self-efficacy in learning English from both groups decreased from pretest to posttest, not supporting H3.

As reminded by Liu et al.’s (2014) meta-analysis, the effectiveness of mind mapping varied across contexts, and the positive impact was not guaranteed. There are three viable explanations for the non-significant results in self-efficacy in learning English and academic performance in English language tests. Firstly, in most studies using mind maps, teachers were intensively engaged in the design and implementation of the mind maps. Often they provided feedback to students’ self-assessment as demonstrated in the mind maps and used the generated insights for subsequent instruction. For the current study, students were required to perform “self-reflection” and “feedback-seeking” (i.e., identify their strengths and weaknesses in the lesson and identify additional resources for help and feedback). Since we did not ask the teacher to review the mind maps or provide feedback to students’ self-assessment for the sake of minimizing teacher workload, there was no monitoring system to ensure that students’ roles were fulfilled as expected. Teachers’ involvement and feedback in this regard could be deemed useful in enhancing student learning (Miller and Geraci, 2011). Though the approach to minimize teacher workload makes our intervention more likely to be sustainable, this is probably with a price of reduced impact on student learning. Secondly, most studies (e.g., Lan et al., 2015; Fu et al., 2019) showing the significant effect of mind mapping on learning the English language shared one common feature: they used tests specifically designed for the studies and focused on the targeted teaching content. The English language pretest and posttest scores used in this study were collected from regular school-based assessments, instead of specifically designed tests. The unfocused tests may blur the real impact of the mind mapping strategy. Thirdly, the intervention period in this study was relatively short (i.e., 8 weeks). As reported in Liu et al.’s (2014) meta-analysis, the time students are exposed to the mind mapping is important in determining the effectiveness of the mind mapping strategy. Mind mapping interventions lasting for 3–6 months were substantially more effective than those lasting for only 1–2 months. This was because the link between mind mapping interventions and observable learning improvements might not be direct, but mediated by other factors. A likely mechanism is that the intervention invokes motivational predictors, such as self-efficacy, which are inner drives of student learning. The inner drives have to result in high-quality behavioral engagement in learning and the use of adaptive learning strategies which, in turn, lead to learning improvements (Skinner, 2016). Each link in this chain takes time.

### Implications and Future Directions

The current study aimed to design a simple and relatively cost-effective instructional intervention and test its effectiveness in improving students’ academic performance, self-efficacy in creativity and learning in the English learning context. The results identified a desirable pattern: students using self-assessment mind maps had larger positive changes on self-efficacy in creativity (with statistical significance) and academic performance. Note that many factors have the potential to enhance the intervention effect, such as teacher assessment literacy (Wu et al., 2021a), teacher feedback (Yang et al., 2021), and school support (Yan, 2021). Since this intervention was intentionally designed with a minimum requirement of teacher workload, those factors have not been manipulated in this study. Given the promising finding, the intervention is worthwhile to further trials in classrooms. If future studies could duplicate similar results of this study, it will provide further credentials to the self-assessment mind map as a sustainable intervention. It could be reasonable to expect a more significant impact if future studies use more teacher input, such as feedback on the mind maps, or a longer intervention period.

Despite the theoretical benefits of the self-assessment mind map strategy, appropriate design and implementation are crucial to bringing its benefits into practice. One of our observations was that it is crucial but challenging to maintain students’ interest in completing the mind maps. The quality of students’ mind maps decreased, as indicated by the number of words and the complexity of drawings in the mind maps, when approaching the end of the intervention. Even though we designed two different mind map templates, students lost their freshness after three or four attempts. Teachers need to consider this issue in designing and using self-assessment mind maps in future. The mind maps should be attractive to students taking into account various factors, such as the subject areas, learning topics, and student characteristics.

Another relevant direction is to apply the mind mapping strategy with the support of digital technologies. Scholars (e.g., Liu et al., 2014; Hazaymeh and Alomery, 2022) suggested that applying mind mapping using software or an application led to more learning gains. It is likely because digital technologies make mind mapping an interactive fun-filled activity that enhances students’ motivation and satisfaction (Rosba et al., 2021). Another advantage of digital mind mapping is that it facilitates collaborative work in small groups (Wang, 2019) so that the idea exchange and brainstorming among group members can further stimulate students’ creativity. Also, collaborative mind mapping is likely to be more effective in enhancing students’ academic performance and self-efficacy (Zheng et al., 2020).

### Limitations

This study has its limitations mainly related to the sample size and characteristics. Firstly, although the two groups were randomly assigned to conditions, students were not randomly assigned to groups. It turned out that there were substantial differences in the baseline performances between the two groups on the English language test. The non-equal baseline performances might influence the intervention effect and, therefore, the interpretation of the results should be cautious. Secondly, the sample size for each group was small, which reduced the statistical power to detect significant differences. Future studies need to consider applying randomized controlled trials design with larger sample sizes.

## Conclusion

As a response to the call for sustainable interventions for developing higher-order abilities, this study presents a simple, relatively cost-effective instructional intervention, i.e., self-assessment mind maps, and examines its effect in a naturalistic learning environment. This intervention synergizes the principles of mind mapping and self-assessment and requires minimum teacher workload. The results supported Hypothesis 1, i.e., self-assessment mind maps enhanced students’ self-efficacy in creativity. It is possible that self-assessment mind maps, on the one hand, developed a sense of control in students by offering flexibility and autonomy in the creation process. On the other hand, it provided personal and contextual scaffolds for the formulation of students’ self-efficacy in creativity. However, the intervention did not improve students’ self-efficacy and academic performance in English learning, not supporting Hypotheses 2 and 3. The non-significant results may be attributed to the absence of teacher feedback to students’ mind maps, the lack of a specifically designed test focusing on the targeted teaching content, and the short intervention period. We urge future studies to test this intervention further using the randomized controlled trials design with larger sample sizes. With the limitations in mind, the findings of this study shed light on designing sustainable instructional strategies for empowerment in creativity.

## Data Availability Statement

The raw data supporting the conclusions of this article are available on request to the corresponding author.

## Ethics Statement

The studies involving human participants were reviewed and approved by Human Research Ethics Committee, The Education University of Hong Kong. Written informed consent to participate in this study was provided by the participants’ legal guardian/next of kin.

## Author Contributions

All authors listed have made a substantial, direct, and intellectual contribution to the work, and approved it for publication.

## Conflict of Interest

The authors declare that the research was conducted in the absence of any commercial or financial relationships that could be construed as a potential conflict of interest.

## Publisher’s Note

All claims expressed in this article are solely those of the authors and do not necessarily represent those of their affiliated organizations, or those of the publisher, the editors and the reviewers. Any product that may be evaluated in this article, or claim that may be made by its manufacturer, is not guaranteed or endorsed by the publisher.
