# Supplementary material for: Enhancing Students’ Self-Efficacy in Creativity and Learning Performance in the Context of English Learning: The Use of Self-Assessment Mind Maps
Source: Front Psychol. 2022 May 11;13:871781. doi: 10.3389/fpsyg.2022.871781 (PMC9131007; doi:10.3389/fpsyg.2022.871781)
Supplement: Supplementary file 1 [file Presentation_1.pdf]

Appendices

Appendix A: Experience in Learning English Questionnaire (English Version)

The following are statements about your experience in learning English. Please fill in an appropriate circle to indicate the degree of your agreement with each statement.

	Strongly Disagree	Disagree	Slightly Disagree	Slightly Agree	Agree	Strongly Agree
Self-efficacy in creativity						
1. I am good at coming up with new ideas during English class.	○	○	○	○	○	○
2. I have a good imagination during English class.	○	○	○	○	○	○
3. I have a lot of good ideas during English class.	○	○	○	○	○	○
4. I am good at coming up with new ways in learning English.	○	○	○	○	○	○
5. I am good at coming up with personally meaningful ways in learning English.	○	○	○	○	○	○
Self-efficacy in learning English						
6. Compared to other students in this class I expected to do well. ………………………………………………	○	○	○	○	○	○
7. I am certain that I can understand the ideas taught in class. ………………………………………………	○	○	○	○	○	○
8. Compared with others in this class, I think I am a good student.	○	○	○	○	○	○
9. I am sure I can do an excellent job on the assignments and homework.	○	○	○	○	○	○
10. I think I will receive good grades in my exams.	○	○	○	○	○	○
11. My study skills are excellent compared with others in this class.	○	○	○	○	○	○
12. I know that I will be able to learn the materials for the tests and exams.	○	○	○	○	○	○

Appendix B: Experience in Learning English Questionnaire (Chinese Version)

以下題項描述你學習英文時的情況。請根據你對下列描述的同意程度，填滿適當的圓空格。

	非常不同意	不同意	略不同意	略同意	同意	非常同意
創意自我效能						
1. 在英文堂上，我擅長產生新的想法。	○	○	○	○	○	○
2. 在英文堂上，我有很好的想象力。	○	○	○	○	○	○
3. 在英文堂上，我有很多好的想法。	○	○	○	○	○	○
4. 我擅長想到新的方法來學習英語。	○	○	○	○	○	○
5. 我擅長想到對自己有效的方法來學習英語。	○	○	○	○	○	○
英文學習自我效能						
6. 我預期我會比同班同學學得更好。	○	○	○	○	○	○
7. 我明白課堂上所教的一切。	○	○	○	○	○	○
8. 和同班同學比較，我是一位成績好的學生	○	○	○	○	○	○
9. 我可以肯定我的堂課和功課都做得十分出色。	○	○	○	○	○	○
10. 我相信我會獲得好成績。	○	○	○	○	○	○
11. 我的學習方法比同班同學較佳。	○	○	○	○	○	○
12. 我知道我能夠學懂教材的內容以應付測驗和考試。	○	○	○	○	○	○

Appendix C: Mind map templates

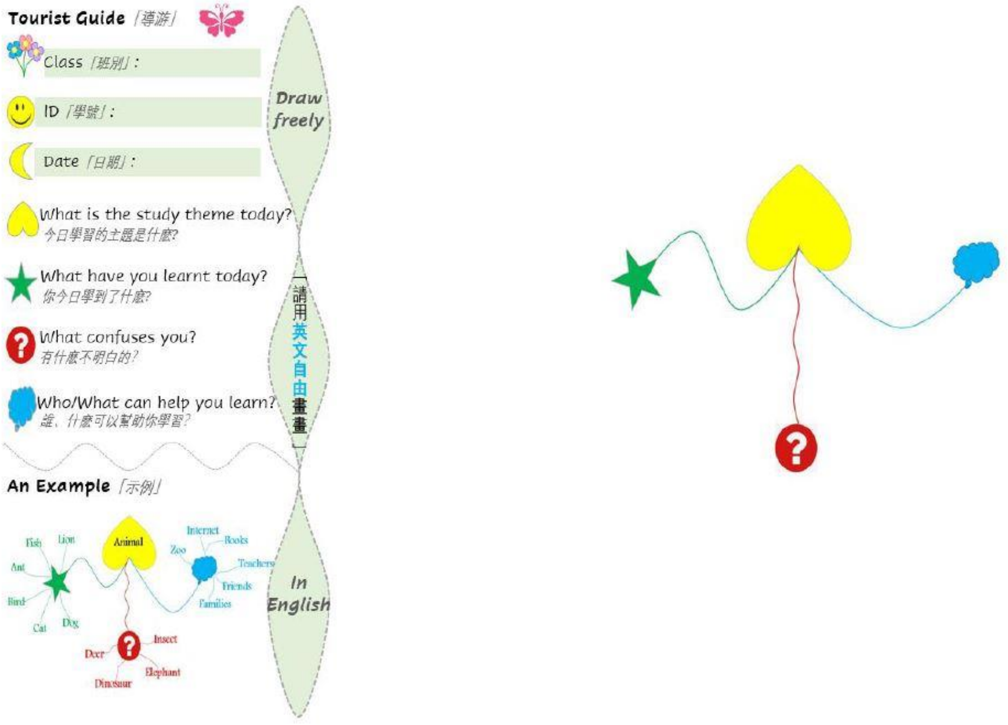

Version 1

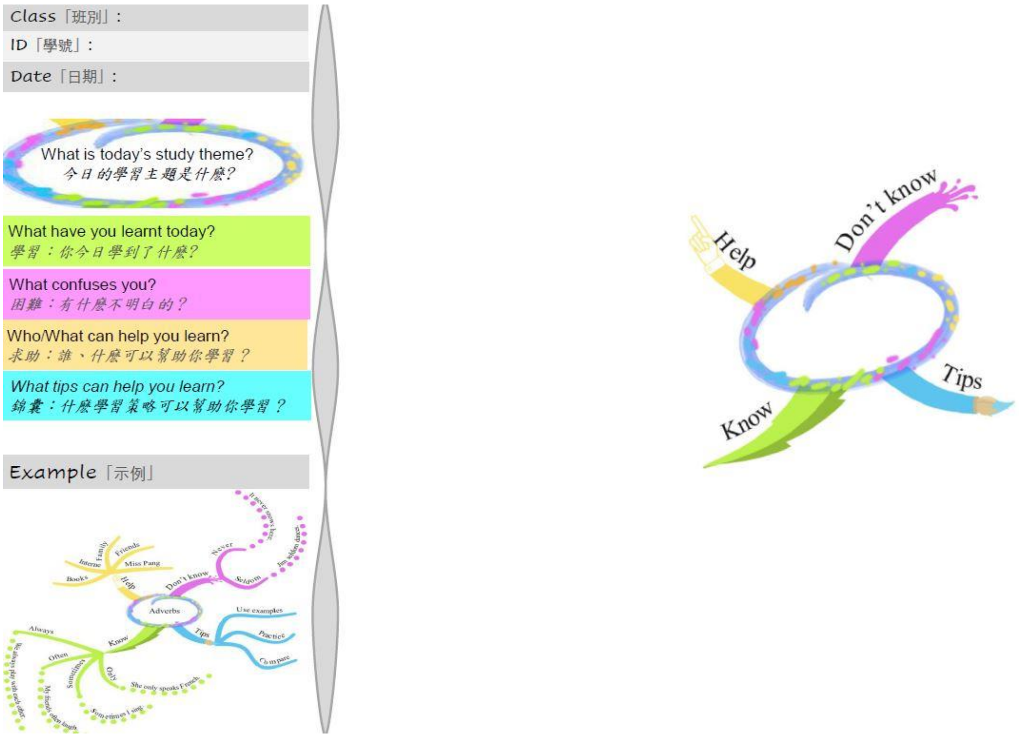

Version 2

Appendix D: The item correlations and item-level statistics for the two scales

For the *self-efficacy in creativity* scale, item correlation ranged from 0.44 to 0.78. and item-total correlations ranged from 0.64 to 0.80. The "Cronbach's alpha if item deleted" were not higher than the Cronbach's alpha of the scale. Each item had a similar mean and standard deviation. The results indicated a high level of internal reliability among these items. For the *self-efficacy in learning English* scale, item correlations ranged from 0.15 to 0.74, and the item-total correlations ranged from 0.44 to 0.77. The lowest correlation was between Items #6 and #7 (r = 0.15). However, the correlations between Item #6 and other items were moderate, ranging from 0.33 to 0.45. The correlations between Item #7 and other items ranged from 0.32 and 0.51. Furthermore, the "Cronbach's alpha if item deleted" did increase if either item was dropped. In addition, both items loaded onto their respective latent constructs (0.404 and 0.749 respectively). Thus, all items were kept.

Table D1 | Item correlations for the *self-efficacy in creativity* scale

	Item 1	Item 2	Item 3	Item 4	Item 5
Item 1	1.00	0.70	0.65	0.55	0.71
Item 2		1.00	0.78	0.44	0.62
Item 3			1.00	0.64	0.57
Item 4				1.00	0.63
Item 5					1.00

Table D2 | Item correlations for the s*elf-efficacy in learning English* scale

	Item 6	Item 7	Item 8	Item 9	Item 10	Item 11	Item 12
Item 6	1.00	0.15	0.45	0.37	0.33	0.41	0.38
Item 7		1.00	0.51	0.32	0.34	0.46	0.47
Item 8			1.00	0.60	0.52	0.57	0.53
Item 9				1.00	0.45	0.74	0.41
Item 10					1.00	0.61	0.72
Item 11						1.00	0.59
Item 12							1.00

Table D3 | Item-level statistics for the scales

	Mean	SD	Item-total Correlation	Cronbach's Alpha if Item Deleted
Self-efficacy in creativity (Cronbach alpha = 0.89)				
Item 1	4.34	1.15	0.77	0.86
Item 2	4.22	1.57	0.74	0.87
Item 3	4.32	1.33	0.80	0.85
Item 4	4.40	1.39	0.64	0.89
Item 5	4.66	1.14	0.74	0.86
Self-efficacy in learning English (Cronbach alpha = 0.86)				
Item 6	4.41	1.24	0.44	0.84
Item 7	4.43	1.27	0.46	0.84

Item 8	3.69	1.36	0.72	0.78
Item 9	4.14	1.18	0.67	0.79
Item 10	5.06	1.07	0.60	0.81
Item 11	3.92	1.20	0.77	0.77
Item 12	4.76	1.14	0.69	0.83
